# Abstract of Proceedings of the Buffalo Medical Association

**Published:** 1867-08

**Authors:** T. M. Johnson

**Affiliations:** Sec’y.


ART. II—*Abstract of Proceedings of the Buffalo Medical Association.*

Tuesday Evening, July 2d, 1867.

The meeting was called to order by the President at the usual hour. Members present—Drs. Eastman, Little, Smith, Kamerling, Rochester, Strong, Congar, Wetmore, Lothrop, Gay and Johnson.

Reading the minutes of the last meeting was, by vote, dispensed with.

Drs. G. E. Mackay and M. W. Potter were proposed for membership.

Dr. Rochester reported the following case:

On the 26th of May a gentleman was attacked with what appeared to be the passage of a renal calculus. He had been treated by Dr. Rochester a year before, for what seemed to be that disorder. He was first seen by Dr. Abbott, Dr. Rochester being professionally occupied elsewhere. There was severe pain in the right lumbar region, stranguary, retraction of right testis and vomiting, with marked chill, followed by febrile movement; tongue coated, bowels costive, abdomen tender and slightly tympanitic, urine scanty, highly colored, with acid reaction and copious lateritious deposit. Dr. Abbott injected half a grain of morphine with hypodermic syringe, and directed hot fomentations and diluent drinks. The patient was much relieved and spent a tolerably good night. He was first seen by Dr. Rochester at 11 A. M. May 27th, who confirmed Dr. Abbott's diagnosis, and essentially continued his treatment. In the evening, however, he found that the indications were rather those of localized peritonitis with disease of the vermiform appendix. He had before met with one case of this disorder which simulated renal calculus passage. The case progressed rather favorably than otherwise until the evening of the 31st. At 4 P. M. on that day the patient was quite comfortable. At 7½ P. M. Dr. Rochester found Dr. Gay in temporary attendance. Comatose symptoms had suddenly manifested themselves about 6 P. M., and so strongly did they resemble narcotism that Dr. Gay supposed an overdose of morphine had been accidentally taken. The pupils were contracted to a point. The respiration was stertorous. The nails and lips were livid. The surface was bedewed with moisture, and the extremities were cold. The patient could be roused by slapping and by shaking, but immediately lapsed into insensibility. For the last twenty-four hours one-fourth of a grain of morphine and two grains of quinine had been given every four hours, alternately, with four drops of Norwood's tincture of veratrum viride, and twelve hours previously half a grain of morphine had been hypodermically applied. It was ascertained that there had been no error in the preparation or administration of the medicine. There was one very strong indication that the coma was not from opium. The respiration was forty per minute. Dr. Rochester expressed the conviction that the coma proceeded from exhaustion, induced by shock caused by gangrene and perforation of the vermiform appendix. Death took place June 1st, at 7 A. M. Post mortem examination was made by Dr. Abbott, at 4 P. M. The appendix (preparation exhibited) was found to be hypertrophied, gangrenous, and perforated by a large orifice half an inch from its cæcal attachment. The foreign body had escaped into the abdominal cavity, and was not discovered. The usual evidences of peritonitis were present.

Dr. Rochester said that there were several noteworthy peculiarities to which he would call attention. First; the outset of the attack, simulating, from sympathetic irritation, the passage of a renal calculus. Second; the age of the patient, forty-nine years, as this is mostly a disorder of young people. Third; the deep coma, at the last, instead of the usual jactitation and nervous irritability.—This was the eleventh case that Dr. Rochester had encountered in a practice of about twenty years; he had, perhaps, had more than his share, but he thought the disease was far more common than was usually supposed, and he thought moreover that disease of the appendix was productive of a majority of cases of so called idiopathic peritonitis, particularly in the male sex. It is a prevalent error that this disorder arises from the accidental introduction of a foreign body into a healthy appendix; the appendix is previously diseased. It is liable to a catarrhal inflammation, by which its cavity is dilated, and its walls are sometimes thickened and sometimes attenuated. Into this dilated pouch foreign bodies find access, but these foreign bodies are very rarely what they are called—cherry-stones, beans, raisin-seeds, etc. They are usually composed of hard fæcal matter, deposited around a nucleus, the nucleus in most instances being a minute biliary calculus, or a phosphatic intestinal concretion—sometimes nearly the whole bulk is of cholestrine or of phosphates, but generally layers of fæcal strata, slowly formed, preponderate. The disease is not necessarily fatal. The appendix may become fast to the cæcum, by peritoneal inflammation, preceding perforation, and the abscess may discharge into the intestine, or it may be circumscribed by organized lymph and find exit through the abdominal wall. Dr. Rochester thinks that he has seen one instance of each of these fortunate terminations. Within the past year two operations have been performed in New York, for the relief of this disorder, and both eventuated successfully. One was made by Willard Parker. But the difficulty of positive early diagnosis, and the enormous hazard of the procedure, will prevent a very frequent resort to the knife. In the case reported to-night, this operation might have succeeded; the disease is confined to the appendix exclusively; it could have been removed entire, but this could not be foreseen or conjectured even, and the event might have been just as fatal. In Dr. Rochester's opinion the possibilities of a spontaneous favorable issue are greater than the probability of a successful surgical operation.

Dr. Lothrop said, these cases in which Dr. Rochester's experience had been so remarkable, were always of great interest. He was inclined to the belief expressed by Dr. R, that most cases of idiopathic peritonitis in the male, originated in the vicinity of the appendix vermiformis, and were connected with inflammatory conditions of that process. He thought that most practitioners must have met with cases of pain, and often induration in that region, at first local, but afterwards spreading over the abdomen, giving rise to symptoms often of great severity, many, however, ending in recovery. He could call to mind a most marked case, recently under his care, in which there was great pain, induration and tension in that region, causing great general disturbance, and having some signs of suppurative inflammation, in which he was expecting an opening into the abdomen and consequent general peritonitis. In this case, though there was no sign of escape of pus into the bowel, such as its passage by the anus, yet he thought it was probable. In many, perhaps most cases, he thought the inflammation arose from the presence of a foreign body or fæcal concretion, lodging in the appendix. But yet it seemed necessary to presuppose some unhealthy condition of the appendix, in order that these bodies should either pass into it, or excite destructive inflammation. He had seen one case in which a fæcal concretion of small size was found after death in the process, a half an inch from its opening, yet no signs of its having excited inflammatory action were visible, the death having been caused by some other disease than peritonitis. It might be that foreign bodies could pass into the appendix without always exciting inflammation, otherwise nature would seem to have made a most prodigious blunder, in setting such a dangerous trap.

The cases which Dr. James Jackson speaks of in his Letter to a Young Physician, as "a painful tumor near the cæcum," may have been somewhat of this nature, though less acute than those affections in which suppuration is known to take place, and all ending in recovery.

Dr. Smith mentioned a case of peritonitis in which there was at first all the usual phenomena of general peritonitis. He treated it in the usual manner, and the patient soon improved, and had almost entirely recovered, when she was again taken with the same symptoms, followed by the discharge of a large quantity of pus into the alimentary canal and passed per rectum. Sent the case to the hospital and lost all knowledge of it thereafter. The general disturbance in this case was undoubtedly due to the local inflammation.

Dr. Lothrop exhibited the specimen of fracture of the femur, just above the knee-joint, of which he had spoken at the previous meeting. The specimen showed no trace of the existence of provisional callus, though the bone was removed one month after the fracture, and a certain amount of definitive union had taken place, the fragments not separating till after considerable maceration and handling. There was no osseous formation about the fractured ends externally.

Dr. Strong said that his time had been so occupied that he had not been able to prepare an essay for this evening, but would be prepared at the next meeting.

By vote of the Society the Doctor was excused for not presenting an essay.

Dr. Strong said that he would report, briefly, a case of puerperal fever. A lady was delivered of a still-born child, and the case went on well until the fourth day, when strong febrile action commenced, and with it all the symptoms of puerperal fever. I saw the case with the attending physician on the seventh or eighth day after confinement. When I first saw her the surface of her body was semi-congested and covered with clammy sweat. Pulse about 130; had incipient retching and instantaneous rejection of everything swallowed. Had had a few hours previous a severe chill. The abdomen was tender and tympanitic. The diagnosis was extensive purulent absorption, and the case looked desperate. The treatment adopted at this time was, morphia sulph. gr. $\frac{1}{8}$, every two hours; quinia sulph. grs. 2, every four hours, and a tablespoonful of brandy every hour. Oleum terebinth was kept constantly applied to the abdomen. We thought she would not live until morning, but on seeing her next morning found her pulse rather stronger and less frequent, and a little less tympany and tenderness; continued the treatment during the second day. On the third day added to the above treatment three grains of the bromide of potassium every four hours. Gave beef essence plentifully. After the fourth day the symptoms were much better. On the fifth day she was considered out of danger, and is now nearly well. I had never before seen so doubtful a case recover from this disease. The case is one of much interest, and I hope that the attending physician will make a full report of it.

The Committee on the revision of the Constitution and By-Laws ask for more time, which was granted.

Adjourned. T. M. Johnson, Sec'y.

